# It is time to examine process stream analysis

**DOI:** 10.1155/S1463924681000357

**Published:** 1981

**Authors:** D. P. Manka

**Affiliations:** 1109 Lancaster Avenue Pittsburgh PA 15218 USA


### It is time to examine process stream analysis

**D.P. Manka**

*1109 Lancaster Avenue, Pittsburgh, PA 15218, USA.*

The number of laboratory methods for the analysis of samples from process streams has increased tremendously so that the analytical chemist need only review the literature to find one suitable for his application. The literature cites many methods by gas and liquid chromatography, atomic absorption, infra-red, ion chromatography, etc.

The direct application of analytical methods to continuous analysis of process streams has recently grown as rapidly or as profusely as laboratory methods. There are many advantages to continuous analysis for example, increased yield of a pure chemical such as in the fractional distillation of benzene, toluene, and xylene. Here, when the operator finds toluene distilling with benzene, he diverts the distillate to a separate tank so that the pure distilled benzene is not contaminated. But it is difficult to assess when all the pure benzene has been distilled and the results of laboratory analysis on stream samples would be too late to be of any value to the operator.

On-stream analyzers reduce quality variations of manufactured products in the petroleum and chemical industries. There is better yield of the valuable chemical and both operating costs and energy consumption are reduced. Plant capacity is increased and a reduction in standard deviation leads to a more uniform product. Reprocessing of off-specification material is reduced which increases customer satisfaction and ultimately sales. In the petroleum industry on stream analysis contributes to longer catalyst life, less coking, and less distillation flooding.

EPA legislation governing pollution sources, toxic or combustible occupational environments, consumer product quality and safety have also contributed to expanding interest in on-stream analyzers.

Microprocessors are replacing mechanical cams, punched or magnetic tape and logic circuit programmers in many designs and following improved reliability and flexibility, more complex sequences are possible. Also, they are well-suited to handling signal manipulations associated with identifying and measuring peak heights and peak areas of detector outputs, compensating for drift, providing automatic calibration to accommodate changing background substances, alarming measurements outside established limits, and rejecting signals caused by spurious transmission noise. Flexibility exists in the form of output that can be specified, and these instruments are easily interfaced with the plant computer for monitoring or control.

In representative distillation column applications, the chromatograph-based control has been found to reduce energy requirements by 10-20%. Other benefits include a 5-10% throughput increase, a 5-30% rise in recovery of the more valuable product, upgrade of the less valuable product and smoother operation of the fractionator or downstream equipment.

The potential returns from optimising catalytic crackers are high because units not only incorporate feed heaters which consume significant quantities of fuel gas, but are large producers and users of steam. For example, in one two-unit complex, an analyzer-based information system was critical in implementing changes which reduced gas consumption by about 100 million BTU per hour, generating a return of over $1 million annually.

The chemist or chemical engineer must determine the advantages of continuous analysis before the installation of the procedure is considered.

This summary is intended to give the chemist or chemical engineer a guide as to the problems encountered in some of the installations and their solutions.

### Sampling point

The site of the analyzer and sampling point should be as close together as possible to decrease the lag time between sampling, analysis, and output of results. The sample may require heat to maintain the constituents and water in vapour form from the sample point to the analyzer. This includes heating the filter which normally must be used to remove the +1.0 micron particles, the Pall Trinity Epocel Filter, MCS 4463 EC is suitable.

If the pressure is high in the plant stream pipe, it should be reduced to about 5 psig, immediately after the sampling point.

The best sampling point is a straight and level section of the plant pipe after the gas or liquid has been mixed several times in the elbows. The exact point should be located at least seven pipe diameters downstream from the last elbow so that there is no further distortion of the flow pattern.

### Sampling probe

The retrieval of a representative sample of gas or liquid is as important as the selection of the analyzer. The analysis is only as good as the sample fed into it. To obtain a representative gas or liquid sample, it is best to sample across the full diameter of the plant pipe at the centre of the pipe and at right angles to the gas flow. It is not advisable to sample from the bottom of the pipe since it generally contains condensed liquids in the case of gases and many solids in the case of liquids.

A good probe is a Teflon tube [1] used to sample coke oven gas which contains fine particles of tar and solid naphthalene. This is a Teflon tube, 1¼-inches OD and ¾-inch ID, which extends across the full width of the plant gas duct. The tube is inserted inside a 2-inch diameter stainless steel pipe with one-half of the pipe cut along the length of the pipe except at the top and bottom so that the six ⅜-inch openings in the Teflon tube are exposed to the gas. The probe is positioned so that the openings are on the same course as the gas flow which causes the tar and naphthalene to be deflected away from the openings as the gas flows past the probe. This probe has been used for eight years without plugging. Steam is connected to the probe for periodic cleaning. The purpose of this sampling probe is for the analysis of $H_2S$, COS, $CS_2$, and HCN in the sour and sweet coke oven gas [1].

### Selection of analyzer

Not all analyzers are suitable for plant installation. Consider the atmosphere in which the analyzer must be located. If $H_2S$ or $SO_2$ is present, then all electrical connections and switches must be operable for a long time in the presence of these gases. Dust and heat are a problem. Air-conditioning and air-pressurising of the analyzer room may be necessary. In modern installations, the analyzer is located in a trailer which can be pressurised and air conditioned. Since the trailer can be locked, unwanted visitors are kept out. It is uncanny how many operators from other sections of the plant are prone to visit a new installation and tinker with valves and switches on an analyzer.

When the method of analysis is determined, be it by gas, liquid, or ion chromatography, atomic absorption, infra-red, etc, inspect the various analyzers available from the manufacturers. Check that the electrical contacts will withstand the action of sulphur gases, dust, and heat. The electrical components should be located in an air purged compartment, preferably away from the analytical sections, so that gas or liquid leaks do not form an explosive mixture. The metals of construction are also important since the analyzer will be subjected to the plant atmosphere for several years.

The standard industrial accuracy of process analyzers is ±1.0%. Reproducibility of ±¼ to ±½% is common. The process analytical system is only as good as the standard used to calibrate the analyzer.

### Gas analysis by infra-red

The easiest and most continuous method for the analysis of $CO_2$, CO, $CH_4$ in blast furnace and basic oxygen furnace top gases and other gas components is infra-red. Gases analyzed by gas chromatography are semi-continuous. However, infra-red analyzers are subject to variations in flow rate, temperature, and atmospheric pressure. For example, CO at 10.2% concentration increases to 10.9% when the room temperature is increased from 22.5°C to 48°C – a typical room temperature increase when the analyzer room is not air conditioned. A similar gas at 0.7% increases to 0.8% under similar temperature increases. At a constant pressure of 2 psig $CO_2$ increases from 13.97% to 14.45% when flow rate is increased from 1.25 scfh to 3.13 scfh. Similarly CO increases from 23.05% to 24.15% under the same conditions. However, hydrogen shows little change in a thermal conductivity cell. Similarly, CO and $CO_2$ concentrations vary with changes in barometric pressure, increasing at increased barometric pressure and decreasing at lower pressure. There are more molecules of $CO_2$ per cubic foot at the higher pressure; therefore, there is a higher concentration.

The best solution to this problem is to heat the gas to 50°C in a special insulated box where the temperature is thermostated to ±0.1°F (0.06°C). The change in flow rate and atmospheric pressure is controlled by absolute pressure regulators located in the same heat controlled box. The gas is preheated in a coil to 50°C and the pressure reduced to 15 psig by means of a 40-E-15 pressure regulator [2]. Excess gas flows from the side of the regulator to atmosphere. Sample pressure is further reduced to 2 psig in a 43-20 absolute pressure regulator [2]. Excess gas is vented to atmosphere from the side of the valve. The pressure is constantly compared to the absolute vacuum section of the valve. This comparison maintains a constant pressure of 2 psig regardless of variation in the pressure of the incoming sample gas. The sample then flows through a small rotometer set at 4 cfh into the $H_2$ cell, the $CO_2$ cell and finally the CO cell. After analysis, the gas flows from the CO cell into the discharge end of another 43-20 absolute regulator [2] in the pressure control box.

Instrument air, at a pressure of 15 psig, is fed to a coil in the pressure control box where it is preheated to 50°C before it is reduced to 2 psig in the second 40-E-15 pressure regulator. Excess air bleeds to atmosphere. The air flows to the inlet of the second 43-20 absolute pressure regulator where it is reduced to 1.5 psig. Air from the inlet and the sample gas from the discharge end of this 43-20 absolute pressure regulator combine within the regulator and together bleed to atmosphere through the side opening of the valve. Constant comparison of the sample gas pressure with the absolute vacuum built into the regulator maintains a constant flow and a constant gas pressure regardless of changes in atmospheric pressure. The maximum pressure maintained in the analyzer is about 0.1 to 0.2 psig higher than the highest atmospheric pressure recorded at the local weather bureau during the past five yesrs. In two such installations, the flow rate and pressure in the analyzer system have remained constant for several years using this system of control.

The analyzer room should be air-conditioned to maintain a constant temperature and pressurised with air to maintain the room dust-free.

Infra-red is the best method for analyzing gases if the absolute pressure regulators are built into the system to eliminate fluctuations due to changes in atmospheric pressure. Each gas is analyzed continuously and easily compensated for interferences by other gases and moisture in the gas mixture. The manufacturer of the infra-red analyzer must know the composition of interfering gases and moisture to make proper mixtures in the infra-red reference cell.

### Process analyzers in the petroleum industry

G.F. Erk [3] has 45 analyzers, 87 measuring points and an IBM 1800 computer to follow and control a Sun Oil Hydrocracker complex. It is equipped with a gas chromatograph to separate the hydrocarbons, a distillation analyzer for the initial boiling point and the end point, oxygen analyzer for heater efficiency and regeneration of the catalytic cracker, a moisture analyzer, infra-red analyzer for gases, specific gravity and a hydrocarbon detector.

W.H. Topham [4] has devised a simple analytical system for the analysis of pentane and butane in LPG. All the electrical components of the chromatograph are within the upper air-purged enclosure, completely segregated from the hydrogen carrier gas and sample located in the bottom section. A simple cam-timer operates the sample valve. There is a one sensitivity potentiometer, a zero adjustment, an auto-manual switch and a millivolt recorder. All components are analyzed at the same sensitivity. These devices decrease the over-all cost of a process analyzer. Small bore lines transfer the sample at a flow of 1 scfh. The vaporiser is a normal pressure reducing valve with an external steam coil which is one-tenth the cost of a specially devised vaporiser.

### Control of fractionating tower

S. Maselli [5] controls both overhead and bottom streams of a fractionating tower.

Tray temperatures provide a continuous and fast inferential measurement of overhead composition. The gas chromatographic analyzer provides a highly accurate direct composition measurement. When the two measurements are combined in a temperature-analyzer cascade system, very tight composition control is produced. Utility reduction is brought about by operating the tower with a greatly reduced reflux ratio. It increases throughput and minimises operator attention.

### Analyzing process octane on line

R.M. Clinton [6] *et al* analyze the octane number of gasoline on the process line rather than with the usual single-cylinder internal combustion engine.

Experiments indicated that as the temperature of a fuel/air mixture is raised, several stages of oxidation occur and that certain parameters of these reactions correlate with octane number of the sample. These parameters provide a basis on which to construct an analyzer capable of simple and accurate octane measurement in process control and monitoring applications.

The on-line explosion-proof octane analyzer simulates the partial oxidation reactions that occur during engine measurements, monitors these reactions, then correlates them with octane ratings. Its design employs a simple oxidation principle and minimises maintenance.

A microthermocouple measures the magnitude of the exothermic reaction which occurs when a sample of gasoline is injected into the air stream and passed into the heated reaction chamber. A second microthermocouple located in the reactor block measures only the temperature rise above the reactor's "ambient" temperature. The exothermic oxidation is characterised by either the peak temperature which occurs during a simple reaction and/or the period of time from the injection of the sample to the initiation of the reaction (induction period). These reactions precede actual combustion and are self-initiating and self-extinguishing. The temperature rise and induction periods of these reactions show good correlation to octane number. As octane decreases, reactions become more severe and induction periods become shorter. Laboratory and field tests show that correlation between temperature rise and octane number is closer; therefore, the analyzer measures only this parameter.

The instrumentation is simple. The reactor assembly is an explosion proof housing, consisting of an insulated stainless steel cylinder with a spherical void in the centre. A proportional temperature controller heats the cylinder to within 0.1°C of the control point, which depends on the analyzer range and specific application. Temperature of the area inside of the explosion dome is regulated by another proportional controller at a control point which assures reproducible flash vaporization of the sample during injection. This controller also preheats the incoming air and sample. Every few minutes a sample of gasoline is injected into the controlled air flow stream to the reactor where the microthermocouple detects the exothermic reaction. The output signal from the differential thermocouple detector is converted to current signal for transmission to the programmer where the millivolt signal is converted into a direct octane number for the recorder.

The programmer contains the necessary hardware to control the sample injection, auto-standardization and data-handling functions. This includes electronic circuitry for "peak picking" the maximum temperature and presenting it as trend output of the desired octane span. A sample-and-hold memory circuit retains the maximum signal level when the temperature reaches peak value and then reads this value out on a continuous chart recording which is updated after each injection.

Since it is totally explosion-proof, it can be installed on-line within a refinery.

### Phenol analysis

B.J. Read *et al* [7] describe the photometric analyzer for analyses of phenols found in effluents of coking operations in steel works and in many chemical processes. It is a difficult measurement because of interfering compounds and suspended matter. However, the measurement is simple with a photometric analyzer which has been modified and refined to make the analyzer stable for process use. It has a light source, usually a gas discharge lamp, which radiates at specific wavelengths only (line source) with each line having a very high sensitivity. The light passes through the aqueous sample which is continuously flowing through the sample cell. The light transmitted by the sample is divided by a semi-transparent mirror into two beams, passing through optical filters to the phototubes.

The filter in one beam transmits only radiation at the selected measuring wavelength. The measuring wavelength is selected so that light intensity reaching the detector varies significantly with a change in the desired gas or liquid concentration, colour, etc. The optical filter in the second beam transmits light at a reference wavelength. This reference wavelength is selected so that light intensity reaching the reference detector varies relatively little with a change in the concentration of the sample.

Measuring and reference wavelengths in some applications are selected so that sample constituents not to be measured absorb light to the same extent. In this way the effects of variations in concentrations of these potentially interfering materials are minimised or eliminated.

From the Beer Lambert Law it follows that the energy reaching the detectors varies logarithmically with concentration. Therefore, if the amplifiers are logarithmic, the output signal is linearly proportional to the concentration.

The differential signal system has the advantage that variation in light source intensity, dirt on the cell windows, bubbles or dirt in the sample have no effect on the final output signal.

Photometric measurement of phenols in a waste effluent stream at a measurement wavelength of 289 nanometers, gives a sensitive test for phenols, as phenol and its homologues are strong absorbers at this wavelength under basic conditions. But other materials present also absorb strongly in this region.

The absorbence spectrum of phenol is pH dependent, and it is this dependence that is used in the measurement to prevent interference by substances which are not pH dependent in their absorbance. A system has been designed which continuously draws a fresh sample of the stream through an analytical cell, and injects either acid or base to adjust the pH roughly to about nine (precise pH adjustment is not necessary). Every 10 minutes acid is injected to lower the pH and the analyzer zero position is set by an automatically operated zero circuit. When this is done, the acid is shut off and base (sodium hydroxide) is continuously added to change the sample pH to about nine. This changes the absorbance of phenols but not the interferences. Phenols can be monitored around the clock down to a few parts per million.

### Electrochemical transducers

These [8] are totally self-contained faradaic devices capable of selectively sensing various gases at stack and ambient levels. These gases are $SO_2$, $NO_X$, $NO_2$, $H_2S$, $CH_2O$, CO, and $O_2$. The sensing principle involves the direct electro-oxidation or electro-reduction of absorbed gas molecules at a sensing electrode which results in a current directly proportional to partial pressure of the specific gas in the sample.

Nitrogen oxide concentrations present in a gaseous mixture are rapidly and continuously monitored by measuring the current passing between an inert metallic sensing electrode and a counter electrode, which electrodes are in contact with an aqueous electrolyte solution and at which sensing electrode the oxides are electrolysed. The sensing electrode is composed of an inert metal, whereas the counter electrode is composed of an electroactive material which is capable of being electro-chemically reduced when electrically interconnected with the sensing electrode in the presence of the aqueous electrolyte solution.

$SO_2$ is electro-oxidised. In this apparatus the electroactive electrode is capable of being electrochemically reduced.

These electrochemical transducers have been applied to power plants, petroleum refineries, smelters, pulp and paper plants, $H_2SO_4$ and $HNO_3$ plants, etc.

### Conclusion

In conclusion, laboratory methods of analyses are well-known for almost every industry. Most of these can be applied directly to a plant stream for continuous analysis. In a few cases, the methods require only a slight modification to make them suitable for stream analyses.

REFERENCES

[1] Manka, D. P., Analysis of $H_2S$ and HCN in coke oven gas, *Instrument Society of America*, 29, part III,701

[2] Pressure and absolute pressure regulators, Moore Products, 2500 Euclid Avenue, Cleveland, Ohio 44117, USA

[3] Eck, G. F., 1970, Performance of a process stream analyser in a Sun Oil complex, *Instrument Society of America,* **25**, 740
[4] Topham, W. H., 1970, New approach to gas chromatographic systems for process control, *Instrument Society of America,* **25**, part III, 749
[5] Maselli, S., 1972, Control both ends for profit, *Instrument Society of America,* **27**, part IV, 831
[6] Clinton, R. M. and Puzniak, T. J., 1975, *Instrument Society of America,* **22**, 47
[7] Read, B. J. and Williamson, J. A., 1975, On-line analysis, *Control and Instrumentation,* 7, 6, 18-21
[8] US Patents 3 622 489 and 3 622 488, Dynascience Corp, Los Angeles, California, USA

### Evaluation of a programmable analyser – the Vitatron PA800

**Janet M. Smith, I. Fry and A. W. Walker**
*Department of Clinical Biochemistry, St. Luke's Hospital, Guildford, Surrey, UK.*

### Introduction

Versatile analysers capable of measuring serum constituents by different principles are gaining increasing popularity in the clinical laboratory. To compete with or complement large multichannel instruments, they must be efficient in terms of running costs, labour costs and speed as well as producing accurate and precise results. The Vitatron programmable analyser (PA800) was evaluated using methods for frequently requested analyses, aspartate transaminase (AST), total bilirubin, cholesterol and glucose as well as for the measurement of phenytoin by the EMIT* technique. The methods were chosen to permit the instrument to display its versatility regarding measuring principles. The evaluation was carried out for the Department of Health and Social Security and the protocol was based on the recommendations of Broughton *et al* [1].

### The Vitatron PA800

The instrument is the successor to the Vitatron Automatic Kinetic Enzyme System (AKES), a discrete analyser, evaluation reports of which have been published elsewhere [2,3]. In addition to performing kinetic analysis of both enzymes and substrates, the new instrument can also perform endpoint analysis. In addition, the computer is capable of curve fitting which increases the range of analyses which can be performed by the instrument. Externally, the PA800 looks very similar to the AKES but there are several differences in its mode of operation. The principle of the transport mechanism, the movement of coded sample holders in a chain through the system synchronised to the rotation of a ring of eighteen thermostatted glass cuvettes through the dispensing points and the lightpath, is unaltered. However, whereas the coded sample holders for the AKES were supplied in chains of ten those for the PA800 are supplied individually. Unnecessary sample holders are not put through the system which improves the instrument efficiency.

The PA800 uses reagents economically, they are sampled from a compartmentalised reagent tray through stainless steel probes in the sampling head by a water–filled syringe system. The disposable reagent trays have two compartments, a smaller inner compartment for 'starter' reagent and a large one for the diluent reagent. For use, the tray is sited adjacent to the cuvette ring. Three separate probes, one each for sample, diluent and starter, are incorporated into the sampling head. Cleaning solution can also be dispensed from each probe into the cuvettes and, allows three cuvette washes between analyses and as a vibrating mixer for the cuvette contents (when reagent or sample are dispensed into a cuvette). Volumes picked up and dispensed through the probes are selected manually by means of micrometer screws on Hamilton syringes. A stainless steel suction tube, positioned in the sampling head, removes the cell contents, under vaccuum, to a waste unit sited behind the sampling head. The photometer has a quartz-iodine lamp and the wavelength is selected by means of interference filters. Two integrators are built into the photometer. During the measurement of absorbance changes, ie whilst one integrator is performing an integration, the other is transferring the results of the previous integration to the calculator.

The system of audio and visual warnings used in the AKES has been incorporated into the PA800, the audible warning drawing attention to the instrument and the warning lamps directing the operator to the source of the problem. In addition, a temperature warning lamp is illuminated if the cuvette ring is not at the programmed temperature.

The analytical process is controlled by the Canon Canola SX - III calculator, which is an integral part of the system. Instructions are fed into the calculator via magnetic cards. Two general program cards are fed in daily. One or more method cards, containing instructions specific for the assay to be performed, including temperature of assay, incubation times, number of measuring points and mode of calculation, are fed in before performing an assay. Information and results are printed on a tally roll. When a method program card has been loaded, the syringe settings and interference filter required are printed by the calculator and adjustments are made manually. Alteration of the diaphragm position on the photometer is also performed manually, but blanking is carried out automatically by the instrument.

The mathematical analysis of the absorbance data for kinetic measurements on the PA800 is more sophisticated than that on the AKES. The photometer integrates the absorbance change over consecutive intervals of 0.287 seconds, the number of readings being programmed for the method in question. These measuring points are divided into four blocks and the sum of integrations within each block is calculated. After the monitoring period, the slope of the line is calculated at two points during the time course of the

** EMIT is a registered trademark of the Syva Corporation.*

